# Prognostic Breast Cancer Signature Identified from 3D Culture Model Accurately Predicts Clinical Outcome across Independent Datasets

**DOI:** 10.1371/journal.pone.0002994

**Published:** 2008-08-20

**Authors:** Katherine J. Martin, Denis R. Patrick, Mina J. Bissell, Marcia V. Fournier

**Affiliations:** 1 Bioarray Consulting, Belmont, Massachusetts, United States of America; 2 Department of Oncology-Biology, Oncology Center of Excellence for Drug Discovery, GlaxoSmithKline, Collegeville, Pennsylvania, United States of America; 3 Cancer Biology, Lawrence Berkeley National Laboratory, Berkeley, California, United States of America; Victor Chang Cardiac Research Institute, Australia

## Abstract

**Background:**

One of the major tenets in breast cancer research is that early detection is vital for patient survival by increasing treatment options. To that end, we have previously used a novel unsupervised approach to identify a set of genes whose expression predicts prognosis of breast cancer patients. The predictive genes were selected in a well-defined three dimensional (3D) cell culture model of non-malignant human mammary epithelial cell morphogenesis as down-regulated during breast epithelial cell acinar formation and cell cycle arrest. Here we examine the ability of this gene signature (3D-signature) to predict prognosis in three independent breast cancer microarray datasets having 295, 286, and 118 samples, respectively.

**Methods and Findings:**

Our results show that the 3D-signature accurately predicts prognosis in three unrelated patient datasets. At 10 years, the probability of positive outcome was 52, 51, and 47 percent in the group with a poor-prognosis signature and 91, 75, and 71 percent in the group with a good-prognosis signature for the three datasets, respectively (Kaplan-Meier survival analysis, p<0.05). Hazard ratios for poor outcome were 5.5 (95% CI 3.0 to 12.2, p<0.0001), 2.4 (95% CI 1.6 to 3.6, p<0.0001) and 1.9 (95% CI 1.1 to 3.2, p = 0.016) and remained significant for the two larger datasets when corrected for estrogen receptor (ER) status. Hence the 3D-signature accurately predicts breast cancer outcome in both ER-positive and ER-negative tumors, though individual genes differed in their prognostic ability in the two subtypes. Genes that were prognostic in ER+ patients are AURKA, CEP55, RRM2, EPHA2, FGFBP1, and VRK1, while genes prognostic in ER− patients include ACTB, FOXM1 and SERPINE2 (Kaplan-Meier p<0.05). Multivariable Cox regression analysis in the largest dataset showed that the 3D-signature was a strong independent factor in predicting breast cancer outcome.

**Conclusions:**

The 3D-signature accurately predicts breast cancer outcome across multiple datasets and holds prognostic value for both ER-positive and ER-negative breast cancer. The signature was selected using a novel biological approach and hence holds promise to represent the key biological processes of breast cancer.

## Introduction

Breast cancer ranks as the second leading cause of death among women with cancer in the US. Early detection of breast cancer has a significant impact on patient survival, though a portion of patients still relapse and rapidly develop a more aggressive form of disease [1]. The identification of individuals with a high risk of relapse has become a primary focus of cancer research. Key steps are determining which patients will benefit from standard care therapies and assessing their chances of disease progression. Accurate identification of high-risk genes may not only lead to the identification of groups of high-risk patients, but also to the discovery of novel therapeutic molecular targets.

Several large studies have been performed to identify predictive gene-signatures and have shown the value of incorporating these signatures to evaluate clinical prognosis in breast cancer [2], [3]. Most gene-signatures have been selected using supervised methods applied to training sets of about 50–100 patients, and then confirmed in larger related sets ranging from 100–300 patients. Surprisingly little overlap has been between the individual genes that comprise signatures identified in different studies. Investigations addressing this lack of overlap, have found that predictive signatures are highly dependent on the specific set of patients that make up the training set [4]. Such disparity in signatures is not limited to breast cancer, but also has been found in schizophrenia studies. Less well studied is whether a given predictive signature that has been identified using a given dataset is also predictive in additional unrelated datasets.

Two predictive signatures for breast cancer identified by microarray analysis have been further developed into clinical multi-gene panel tests [5]. MammaPrint became the first test approved by the FDA for predicting breast cancer relapse and is composed of 70 genes. Oncotype DX, a prognostic test for ER positive breast cancers, has been commercially available since 2004 and is composed of 21 genes. The 70-gene signature was identified by analyzing the large NKI dataset of van de Vijver et al. Unfortunately, subsequent analysis found that this signature did not predict outcome as well in an independent dataset [6]. Several clinical trials are ongoing to test the utility of these prognostic gene-signature tests [7].

Even though gene signatures so far have been helpful for identifying patients at risk, they provide limited information on which genes are relevant to breast cancer biology. It follows that all genes included in gene-signatures cannot be key biological players in cancer progression. We hypothesize that the ability of a signature to demonstrate predictive power across different independent datasets tends to support the conclusion that it is composed of key, biologically relevant genes. The development of novel, biologically-based gene selection approaches may help to find these genes. We applied an unsupervised approach that is not dependent on the composition of a training set. The approach is based on a well-studied and biologically relevant model system that mimics cellular characteristics of human mammary gland. Since the genes are selected based on a biological parameter, they hold promise to represent key biological processes of cancer.

To select a prognostic signature, we used a 3D culture model of non-malignant human mammary epithelial cells (HMEC) [8]. When cultured in laminin-rich extracellular matrix, non-malignant HMEC reacquire the ability to form acini-like structures presenting a hollow lumen, basal polarity and cell cycle arrest. These structures recapitulate many of the characteristics of luminal cell differentiation in the mammary gland [9], [10]. We hypothesized that gene expression associated with acini formation are opposite from those occurring during the development of breast tumors with a poor prognosis. Here we describe the predictive power of a small set of 22 genes that were down-regulated during growth arrest and acini formation of HMEC in 3D cultures (3D-signature) in three large, independent breast cancer microarray datasets.

## Materials and Methods

### Dataset sources

**Figure 1:**
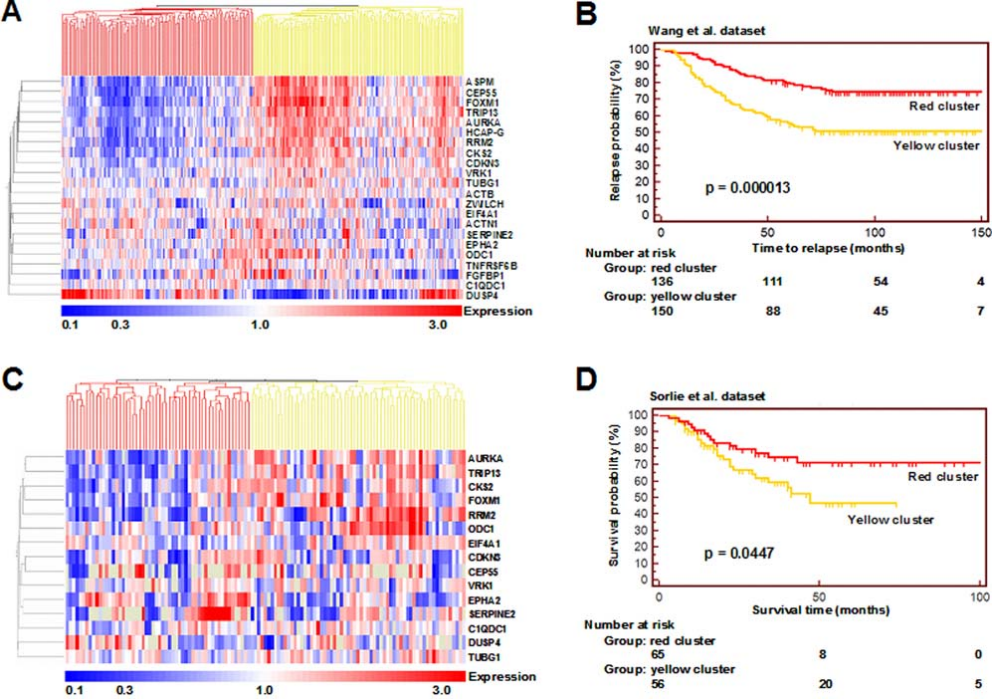
The 22 gene 3D signature predicts survival in the microarray datasets of Wang, *et al.*, and Sorlie, *et al.* The 22 gene signature and unsupervised hierarchical clustering grouped breast cancer patients to accurately reflect overall relapse or survival when analyzed by the method of Kaplan and Meier. A. Hierarchical cluster analysis of the dataset of Wang, *et al.* The pattern of expression of the 22 genes selected by the 3D assay are shown for the 286 breast cancer patients of Wang, *et al.* Genes and samples were organized by using hierarchical clustering. The two major clusters in the sample dimension (red cluster and yellow cluster), were found by using survival analysis to distinguish between good and poor prognosis patients (p<0.0001). B. Kaplan-Meier curves for the red and yellow clusters of the hierarchical diagram of panel A. The endpoint recorded for this dataset was relapse, measured in months. C. Hierarchical cluster analysis of Sorlie, *et al.* dataset. The pattern of expression of the 15 of 22 genes with probes on the Stanford microarrays and with data available for at least 40% of patients are shown for the 121 breast cancer patients reported by Sorlie, *et al.* Expression was organized by hierarchical clustering. The two major clusters in the sample dimension (red cluster and yellow cluster), were found by using survival analysis to distinguish between good and poor prognosis patients (p = 0.00447). D. Kaplan-Meier curves for the red and yellow clusters of the hierarchical diagram of panel C. The endpoint recorded for this dataset was death, measured in months.

The van de Vijver dataset, with profiles of 295 human breast tumors and associated clinical data [11], was obtained from Rosetta Inpharmatics (http://www.rii.com/publications/2002/nejm.html). Downloaded log base 2 data were transformed to linear values and uploaded to GeneSpring GX 7.3 (Agilent Technologies), which transforms to and uses natural log (log e) for all functions. The arrays and genes were normalized to the median of chips and genes. All data processing steps were performed using GeneSpring GX 7.3 software. The Wang dataset, consisting of the microarray profiles of 286 human breast tumors with associated clinical data [12], was obtained from GEO (Series GSE2034). The downloaded data were transformed to set measurements less than 25 to 25, chips and genes were median normalized and median polished. The Sorlie dataset, with microarray profiles of 118 human breast tumors, 4 non-malignant breast samples, and associated clinical data [13], was obtained from GEO (Series 4335). These samples represented a number of different platforms; all platforms were translated to a single dataset for analysis. Log base 2 data were downloaded, transformed to linear, uploaded to GeneSpring GX 7.3 and then chips and genes were median-normalized. Data for at least 40% of the Sorlie, et al., patients were available for 15 of the 3D-signature genes. The gene CEP55 was the gene with the least complete data that was retained for the analysis. For this gene n = 47. For other genes, more complete data were available. Several genes had complete data, n = 118. The missing patients were not consistent across the dataset. The gray blocks of the diagram of Figure 1C show the patients/genes where data was not available. The incompleteness of this dataset likely had an impact on reducing the significance of the results relative to the two larger and more complete datasets. Data for the first ten patients of Desmedt et al [14] were obtained from GEO record GSE7390.

Hierarchical cluster analysis and PCA were performed using GeneSpring GX 7.3. All metrics were applied to log e transformed gene expression data. Median normalized gene expression levels for the 3D signature for the Sorlie dataset ranged from a minimum of 0.015 to a maximum of 41, with a standard deviation of 1.79. For the Wang dataset, levels ranged from a minimum of 0.047 to a maximum of 23, with a standard deviation of 1.02. The clustering metric for the Wang dataset was a Pearson correlation in the genes direction and a smooth correlation in the sample direction. The Sorlie metric was a Pearson metric in both directions. Metrics that gave the best visual separation of expression patterns were used. PCA was performed on the sample direction using the 275 patients of the dataset of Wang, et al., for which at least 5 years of follow up data were available. GeneSpring GX 7.3 default parameters, including mean centering and scaling, were applied.

### Survival analysis

Kaplan-Meier and Cox proportional hazards analyses were computed using MedCalc version 9.3.9.0 (http://www.medcalc.be/). For Kaplan-Meier analysis of the individual signature genes, patients were stratified into quartiles for expression of each marker and survival curves were computed. For Cox proportional hazards analysis, all variables were entered into the model in a single step. The Chi-squared statistic was used to assess the relationship between time and all covariates in the model and the significance level (p) was <0.05 for all tests reported. For the dataset of van de Vijver etal., overall survival was used as an endpoint and 295 patients were included in the analysis. For the dataset of Wang et al., relapse was used as an endpoint and 286 patients were included in the analysis. For the dataset of Sorlie et al., relapse was used as an endpoint and 118 patients were included in the analysis.

### ER+/− comparison

ER status was clinically determined for the Wang, Sorlie, and van de Vijver datasets. Numbers of patient samples included in this study for the Sorlie dataset were ER negative n = 31, ER positive n = 81. The remaining 9 patients with unknown status were not included. For the Wang dataset, ER negative n = 77, ER positive n = 209. For the van de Vijver dataset, ER negative n = 69, ER positive n = 226. Welch t-tests were performed using GeneSpring GX 7.3 to compare log e transformed expression levels of the 3D signature genes in ER positive versus ER negative tumors samples. A Benjamini and Hochberg false discovery rate multigene correction was applied.

## Results

We have previously used a novel unsupervised approach to identify a set of 22 genes that predict prognosis of breast cancer patients [8]. This signature included genes that were down-regulated during breast epithelial cell acinar formation in 3D cultures in laminin-rich extracellular matrix (3D lrECM). Identities, Affymetrix IDs, GeneBank accession numbers, and biological functions of these genes are tabulated (**Table S1**).

To further assess the utility of this 3D-signature, we have used three large independent breast cancer microarray datasets, which include annotated microarray data and associated clinical information. The dataset of Wang *et al.*, includes data from 286 breast cancer patients while that of Sorlie, *et al.* (Stanford/Norway) includes data from 118 breast cancer patients, and 4 normal breast tissue samples. Our previous studies of the 3D-signature made use of the dataset 295 patients of van de Vijver, *et al.*, but did not evaluate prognostic accuracy [8]. This dataset is further evaluated here. Together these datasets represent a total of 699 patients. Numerous differences exist between the datasets. Most notably, the patients were selected by different institutions using different admittance criteria. Database criteria are tabulated (**Table S2**).

For each of the datasets, we used expression patterns of the 3D signature genes and unsupervised hierarchical cluster analysis to group tumors into classes. The clustering algorithm divided the patients into groups based on the expression patterns of the 3D signature. Patients were not selected based on any clinical parameters at any step. Probes for all 22 genes were present on the Affymetrix HG-U133A microarrays used by Wang, *et al.* Hierarchical cluster analysis using these genes resulted in two distinct main clusters (Figure 1A), which were used for further analysis. Kaplan-Meier analysis was performed using relapse as an endpoint. The two clusters were highly significantly associated with prognosis (p = 0.000013, Kaplan-Meier) (**Figure 1 A, B**). At 10 years, the probability of positive outcome was 50.9 +/− 4.1 percent in the group with a poor-prognosis signature and 74.6+/− 3.8 percent in the group with a good-prognosis signature.

Using the same approach, we also tested the dataset of Sorlie, *et al.* This dataset used Stanford two-color spotted microarrays of varying formats. Data for at least 40% of patients were available for 15 of the 3D-signature genes. Hierarchical cluster analysis using these 15 genes resulted in four main clusters (**Figure 1C**). These were grouped into the two left clusters and the two right clusters for further analysis. A visual inspection of the gene expression patterns for both datasets showed that low expressing (blue) genes tended to be on the left and high expressing (red) genes tended to be on the right. Kaplan-Meier analysis was performed using patient death as an endpoint. Results showed that the two clusters were significantly associated with prognosis (p = 0.045, Kaplan-Meier) (**Figure 1 C, D**). At 10 years, the probability of positive outcome was 46.7 +/− 8.8 percent in the group with a poor-prognosis signature and 71.4 +/− 6.7 percent in the group with a good-prognosis signature.

Probes for 18 genes of the 3D signature genes were present on the microarrays used by van de Vijver, *et al.*, and we have previously shown that hierarchical cluster analysis separated the patients into five main clusters (**Figure S1**). Here we analyze these individually and also group them into the two left clusters and the three right clusters and perform Kaplan-Meier analysis of these clusters using overall survival as an endpoint. For both the five-cluster and the two-cluster analyses, the individual groups were highly significantly associated with prognosis (p = 1.04E-08 and 9.41E-10, for the five-cluster and two-cluster analyses, respectively) (**Figure S1**). At 10 years, the probability of positive outcome was 52.5+/−4.8 percent in the group with a poor-prognosis signature and 90.8 +/−2.3 in the group with a good-prognosis signature.

To test the ability of the method to predict the outcome of newly added individual patients, we tested individual patients from a fourth, unrelated dataset. Ten patients from the dataset of Desmedt, *et al.*, [14] (patients 1–10) were mapped onto the most similar patient profile of the clustered Wang dataset. Both of these datasets include breast cancer biopsy samples analyzed with Affymetrix HG-U133A GeneChips. Mapping was performed using a Pearson correlation metric to compare the expression levels of the 3D signature genes of a new patient with all 286 patients of the previously clustered Wang dataset. Correlation coefficients for the ten patients ranged from 0.53 to 0.75. Four of the ten patients mapped to the good prognosis cluster and six to the poor prognosis cluster. Only one the four experienced a relapse within 10 years of follow up (relapse times were 10.5, 1.2, >17.8, and >16.5 years). Of the six that mapped to the poor prognosis cluster, five relapsed within 10 years (relapse times were 6.0, 15.9, 2.0, 0.5, 1.4, and 1.9 years). Though this study was too small to obtain statistics, it supports the conclusion that the 3D signature and hierarchically clustered data can be used for the prediction of individual patients.

To test the statistical validity of the groups identified by the 3D signature, we have used principle component analysis (PCA). PCA allows for the reduction of data complexity by discovering a number of principal components that define most of the data variability. PCA was applied on the 275 patients of the dataset of Wang, *et al.*, for which at least 5 years of follow up data were available. Nine principle components were identified representing 78.1 percent of the variability (data not shown). The major component, principle component 1, represented 33.5 percent of the variability. PCA scores were calculated by computing the standard correlation between the expression profile vector of each patient sample and the principal component 1 vector. Calculating scores in this manner results in values between −1 and 1. In the case of n = 275, PCA scores greater than 0.0118 are significantly positively correlated with the component, while scores less than −0.0118 are significantly negatively correlated (p<0.05). 61 of the 95 (64%) patients who relapsed within 5 years had a significant positive score and 107 of the 180 (59%) patients who did not relapse within 5 years had a significant negative score. A positive score was strongly associated with relapse (p = 0.00014) and a negative score was strongly associated with relapse-free survival (p = 0.000018) (Fisher's exact tests). These results show that the expression patterns of the 3D signature separate breast cancer patients into statistically significant prognosis groups.

**Figure 2:**
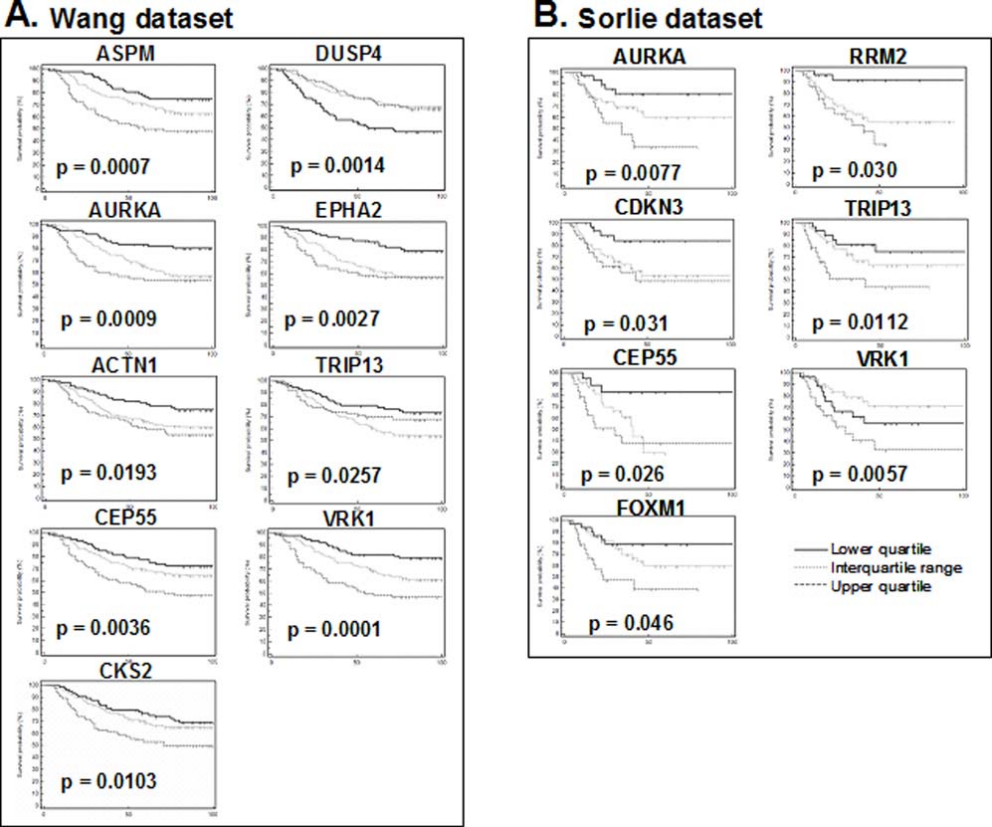
Kaplan-Meier curves of the individual genes that accurately predicted patient prognosis (p<0.05). A. Results for individual genes in the dataset of Wang, *et al.*, using patient relapse as the endpoint. B. Results for individual genes in the dataset of Sorlie, *et al.*, using patient survival as the endpoint.

To begin to address the biology of this system, we tested the ability of each individual gene of the 3D-signature to predict patient survival or relapse . For the Wang dataset, the expression levels of nine genes were significant predictors of a patient's time to relapse (p<0.05, Kaplan-Meier analysis) (**Figure 2A**). These genes included ASPM, AURKA, ACTN1, CEP55, CKS2, DUSP4, EPHA2, TRIP13, and VRK1. For each of these genes except DUSP4, poor prognosis tumors with a short time to relapse were associated with a higher level of gene expression. For DUSP4, the pattern was reversed and poor prognosis was associated with a lower level of expression. For the Sorlie dataset, expression levels of seven genes were significant predictors of survival time (**Figure 2B**). These genes included AURKA, CDKN3, CEP55, FOXM1, RRM2, TRIP13, and VRK1. For all of these genes, poor prognosis was associated with a higher level of gene expression. Kaplan-Meier p-values are summarized in **Table 1**, which also lists our previously determined p-values from the van de Vijver dataset for comparison. The results show that 41% (9 of 22), 39% (7 of 18), and 68% (13 of 19) of the genes were significant individual predictors in the Wang, Sorlie, and van de Vijver datasets, respectively (**Table 1**).

**Table 1:** Kaplan-Meier p-values for the 22 individual 3D signature genes in the Wang, Sorlie, and van de Vijver patient datasets.

Gene	All patients			ER + patients			ER− patients		
	Wang	Sorlie	Van de Vijver*	Wang	Sorlie	Van de Vijver*	Wang	Sorlie	Van de Vijver*
**Cell cycle / mitosis genes**									
ASPM	**0.0007**	-	ns	ns	-	ns	ns	-	ns
AURKA	**0.0009**	**0.0077**	**0.0001**	**0.0062**	0.067	ns	ns	ns	ns
CDKN3	ns	**0.031**	**0.0001**	ns	0.095	ns	ns	ns	ns
CEP55	**0.0036**	**0.026**	**0.0001**	ns	**0.023**	ns	ns	ns	ns
CKS2	**0.01**	ns	**0.05**	ns	ns	ns	ns	ns	ns
DUSP4	**0.0041**	ns	**0.0001**	0.085	ns	ns	ns	ns	ns
NCAPG	ns	-	-	0.087	-	-	ns	-	-
RRM2	ns	**0.03**	**0.0001**	ns	**0.028**	0.068	ns	ns	ns
TUBG1	ns	ns	**0.05**	ns	ns	ns	0.059	ns	ns
**Angiogenesis / motility genes**									
ACTB	ns	-	**0.05**	0.079	-	ns	ns	-	**0.041**
ACTN1	**0.019**	ns	**0.01**	ns	ns	ns	ns	ns	ns
EPHA2	**0.0027**	ns	**0.05**	**0.011**	ns	**0.026**	ns	ns	ns
FGFBP1	ns	ns	ns	ns	ns	**0.049**	ns	ns	0.059
FOXM1	ns	**0.046**	**0.0001**	ns	ns	ns	ns	ns	**0.039**
SERPINE2	ns	ns	-	ns	ns	-	0.092	**0.0088**	-
TNFRSF6B	ns	ns	ns	0.076	ns	ns	ns	ns	0.071
ZWILCH	ns	ns	**0.01**	ns	ns	ns	ns	ns	ns
**Polyamine biosynthesis**									
ODC1	ns	ns	ns	ns	ns	ns	ns	ns	ns
**Transcription / translation genes**									
EIF4A1	ns	ns	ns	0.095	ns	ns	ns	ns	ns
TRIP13	**0.0257**	**0.0112**	**0.0001**	ns	ns	ns	ns	ns	ns
VRK1	**0.0001**	**0.0057**	ns	**0.018**	**0.026**	ns	ns	ns	ns
**Unknown function**									
C1QDC1	ns	ns	-	ns	ns	-	ns	ns	-

* Data previously reported (Fournier et al., Cancer Research 2007).

ns = not significant; - = no data; bold = p<0.05.

**Table 1** groups the 3D-signature genes by the biological process in which they participate. Group assignments were made using information from GO biological process terms, UniProt-SwissProt function, or literature searching. The genes include five categories: cell cycle/mitosis, motility/angiogenesis, polyamine biosynthesis, transcription/replication genes, and one gene of unknown function.

We have also looked at the ability of the individual genes to predict prognosis in ER+ and ER− subsets of patients. **Table 1** lists Kaplan-Meier p-values for ER+ and ER− tumors for all three datasets. A notable finding among the ER related differences was that the genes that tended to associate with prognosis in ER+ patients had different molecular functions than the genes that tended to associate with prognosis in ER− patients. In particular, significantly more cell cycle and transcription genes were prognostic markers for ER+ tumors (Fisher's exact test, p = 0.0047), while prognostic markers of ER− tumors were significantly more likely to have functions related to angiogenesis and metastasis (Fisher's exact test, p = 0.023). This analysis considered results from all three of the datasets. The genes that tended to associate with prognosis in ER+ tumors (p<0.1 for at least one of the three datasets) included AURKA, CDKN3, CEP55, DUSP4, NCAPG, RRM2, ACTB, EPHA2, FGFBP1, TNFRSF6B, EIF4A1, and VRK1 (**Table 1**). Genes that tended to associate with prognosis in ER− tumors included TUBG1, ACTB, FGFBP1, FOXM1, SERPINE2, and TNFRSF6B. Genes that were markers for prognosis in both ER+ and ER− tumors included ACTB, FGFBP1, and TNFRSF6B. We note that caution is needed in interpreting these results as the data set included relatively small numbers of both genes and patients in the various subsets. In particular, the lack of significant Kaplan-Meier p-values among cell cycle and transcription related genes for ER− patients could be explained by the low patient numbers. Ideally, this comparison would have equivalent patient numbers for both ER subsets.

We have also found a statistical association between expression of the individual 3D-signature genes and tumor ER status, though small fold changes suggest this may be of limited biological relevance (**Table 2**). Expression levels of the majority of the 22 genes were significantly associated with ER status. For the Wang, Sorlie, and van de Vijver patient datasets, percentages associated with ER status were 91%, 71%, and 84%, respectively. There was a very strong statistical enrichment for ER status related genes among the 3D-signature genes (Fisher's exact test, p = 3.11E-8, Wang dataset). In the Wang dataset, the expression levels of 20 of the 22 signature genes (91%) were significantly associated with ER status, while, for the entire set of 22,283 genes, expression levels of a total of 7,424 genes (33%) were ER associated (Welch t-test with a false discovery rate multigene correction, p<0.05). The genes that correlated with ER status also correlated with basal/luminal status (Fisher's exact test p = 0.011) (data not shown). The majority of the genes were more highly expressed in ER− breast cancers than ER+ breast cancers. Two genes (DUSP4 and TUBG1) had the reverse pattern and were significantly under-expressed in ER-negative tumors (correlation analysis, p<0.05). In the Wang dataset, we found that the highly ER-associated genes were no more likely to be good prognostic markers than the non-ER-associated genes (Fishers exact tests, p<0.05) (**Table 1**). This conclusion applied to the subsets of ER+ tumors and ER− tumors, as well as all patients. These statistically significant p-values may not be biologically relevant since fold changes were small. Genes with the most significant differences between ER positive and ER negative tumors (e.g. ODC, CEP55, and EPHA2 in the van de Vijver dataset), had approximately 20 percent (1.2-fold) differences in median gene expression levels. Other genes had smaller fold changes.

**Table 2:** ER association of the 22 individual 3D signature genes in three patient datasets (Welch t-test p values with false positive multigene correction).

Gene	Wang	Sorlie	van de Vijver
**Cell cycle / mitosis genes**			
ASPM	3.1e-9	-	9.2e-5
AURKA	3.6e-8	0.018	1.2e-8
CDKN3	3.8e-6	ns	0.024
CEP55	4.5e-10	ns	6.9e-10
CKS2	0.0011	3.8e-9	0.0017
DUSP4	5.3e-7	0.044	6.1e-9
NCAPG	2.4e-5	-	-
RRM2	7.6e-12	0.049	2.7e-9
TUBG1	ns	0.018	ns
**Angiogenesis / motility genes**			
ACTB	0.018	-	5.8e-11
ACTN1	2.4e-5	ns	0.027
EPHA2	1.7e-9	0.028	1.2e-10
FGFBP1	1.6e-6	-	0.00069
FOXM1	1.7e-9	0.025	3.5e-9
SERPINE2	5.1e-5	ns	-
TNFRSF6B	0.0030	ns	0.0017
ZWILCH	0.0081	0.00018	0.0015
**Polyamine biosynthesis**			
ODC1	6.7e-11	ns	5.8e-11
**Transcription / translation genes**			
EIF4A1	0.018	0.018	ns
TRIP13	9.0e-9	0.0042	1.2e-10
VRK1	0.0061	4.2e-5	ns
**Unknown function**			
C1QDC1	ns	0.00094	-

To further assess the predictive capability of the 3D signature while correcting for known clinical risk factors, we performed Cox proportional hazard analysis. Univariable hazard ratios for poor outcome over a period of 10 years were 5.5 (95% CI 3.0 to 12.2, p<0.0001), 2.4 (95% CI 1.6 to 3.6, p<0.0001), and 1.9 (95% CI 1.1 to 3.2, p = 0.016), for the van de Vijver, Wang, and Sorlie datasets, respectively (**Table 3**). For all of these results, 95 percent confidence intervals exclude 1.0 and p-values (<0.05) show statistical significance. The hazard ratios for the 3D signature remained significant for the two larger datasets (p<0.0001 for both van de Vijver and Wang, *et al.*) when a multivariable analysis was performed to correct for ER status. In this multivariable analysis, ER status was a significant factor in only the van de Vijver dataset (p = 0.0044), but not in the Wang (p = 0.32) or Sorlie (p = 0.41) datasets. The smaller dataset of Sorlie, *et al.*, may have had insufficient numbers of patients to achieve a significant result for the 3D signature in the multivariable analysis. This result shows that the 3D signature accurately predicts breast cancer outcome in both ER-positive and ER-negative tumors.

**Table 3:** Univariable and multivariable proportional-hazards analysis of survival risk for three large independent testing sets*.

	Univariable analysis		Multivariable analysis#	
	Hazard ratio (95% CI)^a^	p	Hazard ratio (95% CI)	p
**van de Vijver, et al. dataset**				
ER positive vs negative	0.31 (0.20 to 0.49)	<0.0001	0.50 (0.31 to 0.80)	0.0044
3D signature	5.52 (2.98 to 10.22)	<0.0001	4.45 (2.35 to 8.43)	<0.0001
**Wang, et al. dataset**				
ER positive vs negative	1.00 (0.65 to 1.54)	0.99	1.25 (0.80 to 1.95)	0.32
3D signature	2.40 (1.60 to 3.60)	<0.0001	2.51 (1.66 to 3.80)	<0.0001
**Sorlie, et al. dataset**				
ER positive vs negative	0.69 (0.40 to 1.20)	0.19	0.79 (0.44 to 1.39)	0.41
3D signature	1.89 (1.13 to 3.17)	0.016	1.51 (0.88 to 2.58)	0.13

* Results for the datasets of van de Vijver, *et al.*, and Wang, *et al.*, represent 10 year Hazard Ratios (95%CI). Results for the dataset of Sorlie, *et al.* were calculated using all available data, which included 5 years of follow up. The endpoint for the van de Vijver analysis was overall survival and for the Wang and Sorlie analyses were relapse.

# Multivariable analysis accounted for ER status and the 3D signature.

a CI, confidence interval.

Multivariable analysis shows the overall risk of death predicted by the 3D-signature and six known clinical factors in the dataset of van de Vijver, *et al.* (**Table 4**). Results show that the 3D signature was the strongest independent factor in predicting breast cancer outcome (Hazard ratio = 4.43, 95 percent confidence interval 2.32 to 8.46, p<0.00001). Additional independent predictive factors were age, tumor size, and ER status.

**Table 4:** Multivariable proportional-hazards analysis of 10 year survival risk*.

	Hazard ratio (95% CI)^a^	p
Age (per 10 year increment)	0.62 (0.44 to 0.88)	0.008
Tumor diameter (per cm)	1.33 (1.04 to 1.69)	0.023
ER (positive vs negative)	0.55 (0.34 to 0.90)	0.018
Lymph node status (per positive node)	1.07 (0.96 to 1.20)	0.234
Chemotherapy	0.69 (0.38 to 1.26)	0.234
Mastectomy	1.05 (0.63 to 1.73)	0.864
3D signature	4.43 (2.32 to 8.46)	<0.00001

* Results were calculated using the dataset of van de Vijver, et al. using overall survival as the endpoint. Similar results were obtained for the same multivariable analysis using relapse as the endpoint, 3D signature Hazard ratio 3.3 (95% CI 2.0 to 5.3), p<0.0001.

a CI, confidence interval.

## Discussion

We hypothesized that the changes in gene expression occurring during acini formation of non-malignant HMEC in a 3D culture model are opposite from those occurring during the development of breast tumors with a poor prognosis. In support of this hypothesis, we showed that genes that were expressed at significantly lower levels in organized, growth arrested HMEC than in their proliferating counterparts could be used to classify breast cancer patients into poor and good prognosis groups [8]. The present study provides three independent confirmations of a 22 gene prognostic signature (3D-signature) that we previously identified using a novel unsupervised strategy.

One of the key criticisms of gene signatures identified using microarray technology is the lack of validation across platforms [13], [15]. Here we report that the 3D-signature predicted prognosis in three large independent datasets (p = 1.3E-5, 0.045, and 9.4E-10 for datasets of Wang, *et al.*, Sorlie, *et al.*, and van de Vijver, *et al.*, respectively; Kaplan-Meier analysis). These three large datasets represent a total of 699 breast cancer patients. There were differences in how well the signature performed between the datasets. Prognosis was best for the Wang and van de Vijver datasets. Microarrays used for the Wang dataset were identical to those of our selection study and included probes for all 22 3D signature genes. In contrast, microarrays used for the Sorlie dataset included probes for only 15 of the 22 genes, and some of these 15 probes could potentially recognize different isoforms of the genes than those of the selection study. However, even with these differences in probe composition, the 3D-signature accurately predicted prognosis.

The 3D-signature includes cell cycle and transcription related genes that predict prognosis in ER+ breast cancer patients. This finding is consistent with previous studies that show that proliferation and cell cycle genes are the strongest predictor for relapse among ER positive patients [16]. In several previous studies, a signature enriched in cell cycle related genes has been reported to predict poor prognosis of breast cancer, along with a second smaller class of genes that includes transcription related genes. Poor prognosis in ER+ tumors in particular has been found to be strongly predicted by over expression of cell cycle and cell proliferation genes [12], [17], [18], [19].

The 3D-signature also includes angiogenesis and motility genes that are markers for prognosis in both ER+ and ER− tumors. Genes in this functional class of breast tumor marker genes were also identified in other breast cancer signatures [17], [20], though the association of this functional class with ER− tumors has not been noted for gene signatures. Markers for ER− tumors have been reported to be significantly less prevalent than markers for ER+ tumors [19]. Some genes within this functional class predicted prognosis for only ER+ tumors, some predicted prognosis for only ER− tumors, and some predicted prognosis for both ER+ and ER− tumors.

Since few overlaps have been found among the published breast cancer signatures, it appears that many (thousands) of marker genes have predictive ability in different subsets of patients. It has been proposed that some genes may have moderate predictive ability in many patients, while some may be “master genes” with high predictive ability in as yet undefined subsets of patients [13]. When many such genes are used together, a highly accurate predictive tool results that is accurate across a wide cross section of breast cancer patients. The actual composition of the signature may be less important than the fact the each signature is a set of many semi-predictive genes. In contrast to gene signatures identified from specific patient sets by supervised methods, our approach is based on a well studied and biologically relevant model system that mimics the human mammary gland. Hence the 3D-signature holds promise to include “master genes” of key biological processes of cancer.

Several examples from existing literature support the hypothesis that the 3D signature genes play important biological roles. First, several of the genes identified have been reported to be up-regulated in tumor cell lines, implicated in tumor growth, angiogenesis and/or metastasis in animal models, and are under investigation for development of novel target therapies. The EphA2 receptor tyrosine kinase is frequently over-expressed in aggressive breast cancer and has been associated with breast tumor growth in animal models [21], [22] and resistance to therapy with tamoxifen [23], and thus reduction in its expression is currently being considered as a potential target for therapy [24]. Monoclonal antibodies to down modulate EphA2 and siRNA studies were reported to inhibit the growth of human breast and lung tumor xenografts in nude mice and tumor angiogenesis and metastatic progression [25]. Among the most predictive genes in the 3D signature are aurora kinase A (AURKA) and CEP55. AURKA is a validated therapeutic target for treatment of cancers and there currently are small molecule inhibitors of aurora kinases being evaluated in the clinic. CEP55 has been identified as a regulator required for cell cycle progression and completion of cytokinesis by loss-of-function studies and it is over-expressed in several cancer cell lines [26]. The transcription factor forkhead box M1 (FOXM1) has been shown to be up-regulated in a variety of carcinoma cell lines and its expression is suppressed in terminally differentiated cells. Its up-regulation has been shown to lead to proliferation of tumor cells and the formation of lung tumors in transgenic mice [27], while its down regulation has been shown to lead to the inhibition of invasion and angiogenesis in pancreatic cancer cells [28]. For these reasons, inhibitors of FOXM1 are currently under investigation to develop anticancer drugs [29]. The lesser known gene TRIP13, a thyroid hormone receptor interactor, is a protein that interacts with hormone-dependent transcription factors to regulate the expression of a variety of specific genes, suggesting that it could have a relevant role in breast cancer biology and be a target for development of novel therapeutics.

Earlier detection can benefit patient survival and treatment options; however progress is still needed in developing therapeutic strategies amenable to early stage disease. A focus on the development of novel treatments targeting early disease rather than advanced malignant carcinoma seems to be a natural next step. The identification of key regulatory pathways that maintain the self-limited proliferation of non-malignant cells in 3D cultures may direct us to novel molecular targets for earlier cancer therapy.

## Supporting Information

Table S1 List of 22 genes in the 3D-signature.
(0.05 MB DOC)

Table S2 Comparison of microarray datasets
(0.03 MB DOC)

Figure S1 Kaplan-Meier curves of the individual genes that did not accurately predict patient prognosis (p>0.05). A. Results for individual genes in the dataset of Wang, et al. using patient relapse as the endpoint. B. Results for individual genes in the dataset of Sorlie, et al. using patient survival as the endpoint.
(0.10 MB TIF)

## References

[1] (2007). Cancer Facts & Figures.

[2] Edgren H, Kallioniemi O (2006). Integrated breast cancer genomics.. Cancer Cell.

[3] Acharya CR, Hsu DS, Anders CK, Anguiano A, Salter KH (2008). Gene expression signatures, clinicopathological features, and individualized therapy in breast cancer.. Jama.

[4] Ein-Dor L, Zuk O, Domany E (2006). Thousands of samples are needed to generate a robust gene list for predicting outcome in cancer.. Proc Natl Acad Sci U S A.

[5] Hinestrosa MC, Dickersin K, Klein P, Mayer M, Noss K (2007). Shaping the future of biomarker research in breast cancer to ensure clinical relevance.. Nat Rev Cancer.

[6] Gruvberger SK, Ringner M, Eden P, Borg A, Ferno M (2003). Expression profiling to predict outcome in breast cancer: the influence of sample selection.. Breast Cancer Res.

[7] Branca M (2003). Genetics and medicine. Putting gene arrays to the test.. Science.

[8] Fournier MV, Martin KJ, Kenny PA, Xhaja K, Bosch I (2006). Gene expression signature in organized and growth-arrested mammary acini predicts good outcome in breast cancer.. Cancer Res.

[9] Petersen OW, Ronnov-Jessen L, Howlett AR, Bissell MJ (1992). Interaction with basement membrane serves to rapidly distinguish growth and differentiation pattern of normal and malignant human breast epithelial cells.. Proc Natl Acad Sci U S A.

[10] Bissell MJ (2007). Modelling molecular mechanisms of breast cancer and invasion: lessons from the normal gland.. Biochem Soc Trans.

[11] van de Vijver MJ, He YD, van't Veer LJ, Dai H, Hart AA (2002). A gene-expression signature as a predictor of survival in breast cancer.. N Engl J Med.

[12] Wang Y, Klijn JG, Zhang Y, Sieuwerts AM, Look MP (2005). Gene-expression profiles to predict distant metastasis of lymph-node-negative primary breast cancer.. Lancet.

[13] Sorlie T, Tibshirani R, Parker J, Hastie T, Marron JS (2003). Repeated observation of breast tumor subtypes in independent gene expression data sets.. Proc Natl Acad Sci U S A.

[14] Desmedt C, Piette F, Loi S, Wang Y, Lallemand F (2007). Strong time dependence of the 76-gene prognostic signature for node-negative breast cancer patients in the TRANSBIG multicenter independent validation series.. Clin Cancer Res.

[15] Esteva FJ, Hortobagyi GN (2004). Prognostic molecular markers in early breast cancer.. Breast Cancer Res.

[16] Loi S, Piccart M, Sotiriou C (2007). The use of gene-expression profiling to better understand the clinical heterogeneity of estrogen receptor positive breast cancers and tamoxifen response.. Crit Rev Oncol Hematol.

[17] van't Veer LJ, Dai H, van de Vijver MJ, He YD, Hart AA (2002). Gene expression profiling predicts clinical outcome of breast cancer.. Nature.

[18] Sotiriou C, Wirapati P, Loi S, Harris A, Fox S (2006). Gene expression profiling in breast cancer: understanding the molecular basis of histologic grade to improve prognosis.. J Natl Cancer Inst.

[19] Teschendorff AE, Naderi A, Barbosa-Morais NL, Pinder SE, Ellis IO (2006). A consensus prognostic gene expression classifier for ER positive breast cancer.. Genome Biol.

[20] Chang HY, Nuyten DS, Sneddon JB, Hastie T, Tibshirani R (2005). Robustness, scalability, and integration of a wound-response gene expression signature in predicting breast cancer survival.. Proc Natl Acad Sci U S A.

[21] Fang WB, Brantley-Sieders DM, Parker MA, Reith AD, Chen J (2005). A kinase-dependent role for EphA2 receptor in promoting tumor growth and metastasis.. Oncogene.

[22] Macrae M, Neve RM, Rodriguez-Viciana P, Haqq C, Yeh J (2005). A conditional feedback loop regulates Ras activity through EphA2.. Cancer Cell.

[23] Lu M, Miller KD, Gokmen-Polar Y, Jeng MH, Kinch MS (2003). EphA2 overexpression decreases estrogen dependence and tamoxifen sensitivity.. Cancer Res.

[24] Carles-Kinch K, Kilpatrick KE, Stewart JC, Kinch MS (2002). Antibody targeting of the EphA2 tyrosine kinase inhibits malignant cell behavior.. Cancer Res.

[25] Brantley-Sieders DM, Fang WB, Hicks DJ, Zhuang G, Shyr Y (2005). Impaired tumor microenvironment in EphA2-deficient mice inhibits tumor angiogenesis and metastatic progression.. Faseb J.

[26] Martinez-Garay I, Rustom A, Gerdes HH, Kutsche K (2006). The novel centrosomal associated protein CEP55 is present in the spindle midzone and the midbody.. Genomics.

[27] Wang IC, Meliton L, Tretiakova M, Costa RH, Kalinichenko VV (2008). Transgenic expression of the forkhead box M1 transcription factor induces formation of lung tumors.. Oncogene.

[28] Wang Z, Banerjee S, Kong D, Li Y, Sarkar FH (2007). Down-regulation of Forkhead Box M1 transcription factor leads to the inhibition of invasion and angiogenesis of pancreatic cancer cells.. Cancer Res.

[29] Bhat UG, Zipfel PA, Tyler DS, Gartel AL (2008). Novel anticancer compounds induce apoptosis in melanoma cells.. Cell Cycle.

