# Supplementary material for: Prognostic Breast Cancer Signature Identified from 3D Culture Model Accurately Predicts Clinical Outcome across Independent Datasets
Source: PLoS One. 2008 Aug 20;3(8):e2994. doi: 10.1371/journal.pone.0002994 (PMC2500166; doi:10.1371/journal.pone.0002994)
Supplement: Table S1 — List of 22 genes in the 3D-signature. (0.05 MB DOC) [file pone.0002994.s001.doc]

| **Table S1. List of 22 genes in prognostic signature of Fournier et al. 2006** | | | | |  |
| --- | --- | --- | --- | --- | --- |
| Symbol | Alias | Affymetrix ID | GenBank | Description | Functional class |
| ASPM | FLJ10517 | 219918_s_at | NM_018123 | asp (abnormal spindle) homolog, microcephaly assoc | cell cycle/mitosis |
| AURKA | STK6 | 218542_at | NM_018131 | aurora kinase A | cell cycle/mitosis |
| CDKN3 | - | 218456_at | NM_023925 | cyclin-dep kinase inhib 3 (CDK2-assoc dual spec phos) | cell cycle/mitosis |
| CEP55 | FLJ10540 | 209714_s_at | AF213033 | centrosomal protein 55kDa | cell cycle/mitosis |
| CKS2 | - | 204170_s_at | NM_001827 | CDC28 protein kinase regulatory subunit 2 | cell cycle/mitosis |
| DUSP4 | - | 204014_at | NM_001394 | dual specificity phosphatase 4 | cell cycle/mitosis |
| NCAPG | HCAP-G | 214805_at | U79273 | chromosome condensation protein G | cell cycle/mitosis |
| RRM2 | - | 203499_at | NM_004431 | ribonucleotide reductase M2 polypeptide | cell cycle/mitosis |
| TUBG1 | - | 205014_at | NM_005130 | tubulin, gamma 1 | cell cycle/mitosis |
| ACTB | - | 202580_x_at | NM_021953 | actin, beta | motility / angiogenesis |
| ACTN1 | - | 218663_at | NM_022346 | actinin, alpha 1 | motility / angiogenesis |
| EPHA2 | - | 209773_s_at | BC001886 | EPH receptor A2 | motility / angiogenesis |
| FGFBP1 | HBP17 | 208637_x_at | BC003576 | heparin-binding growth factor binding protein | motility / angiogenesis |
| FOXM1 | - | 203856_at | NM_003384 | forkhead box M1 | motility / angiogenesis |
| SERPINE2 | - | 212190_at | AL541302 | serpin peptidase inhib 2 (nexin) | motility / angiogenesis |
| TNFRSF6B/RTEL1 |  | 200801_x_at | NM_001101 | tumor necrosis factor receptor superfamily, 6b, decoy | motility / angiogenesis |
| ZWILCH | FLJ10036 | 218349_s_at | NM_017975 | zwilch, kinetochore associated, homolog | motility / angiogenesis |
| ODC1 | - | 206467_x_at | NM_003823 | ornithine decarboxylase 1 | polyamine biosynthesis |
| EIF4A1 | - | 201714_at | NM_001070 | eukaryotic translation initiation factor 4A, isoform 1 | transcription/replication |
| TRIP13 | - | 204033_at | NM_004237 | thyroid hormone receptor interactor 13 | transcription/replication |
| VRK1 | - | 204092_s_at | NM_003600 | vaccinia related kinase 1 | transcription/replication |
| C1QDC1 | CAPRIN2 | 200790_at | NM_002539 | C1q domain containing 1 | unknown function |
