# Supplementary material for: Prognostic Breast Cancer Signature Identified from 3D Culture Model Accurately Predicts Clinical Outcome across Independent Datasets
Source: PLoS One. 2008 Aug 20;3(8):e2994. doi: 10.1371/journal.pone.0002994 (PMC2500166; doi:10.1371/journal.pone.0002994)
Supplement: Table S2 — Comparison of microarray datasets (0.03 MB DOC) [file pone.0002994.s002.doc]

Histology subtypes included 4 normal breast, 2 DCIS, 100 invasive ductal carcinoma, 3 fibroadenoma, 8 lobular carcinoma, and 1 each of mucinous, papillary, pleomorphic, and undifferentiated carcinomas

122

10-19

8 different platforms ranging from 9,200 to 54,000 features

Fluorescent arrays made at Stanford University

PNAS 2003; 100:8418-23

**Sorlie**

Lymph-node negative patients with no systemic therapy

286

22

23,000

Affymetrix HG-U133A

Lancet 2005; 365:671–79

**Wang**

Stage I or II invasive carcinoma patients less than 52 years of age

295

19

25,000

Fluorescent array made by Rosetta Inpharmatics

NEJM 2002; 347:1999-2009

**van de Vijver**

**Breast cancer patient population**

**No. samples**

**Signature genes on array**

**No. spot features**

**Array platform**

**Citation**

**First author**

**Patient samples**

**Microarray**

**Publication**

**Table S2. Comparison of microarray datasets.**
