# Supplementary figures and images for: Prognostic Breast Cancer Signature Identified from 3D Culture Model Accurately Predicts Clinical Outcome across Independent Datasets
Source: PLoS One. 2008 Aug 20;3(8):e2994. doi: 10.1371/journal.pone.0002994 (PMC2500166; doi:10.1371/journal.pone.0002994)

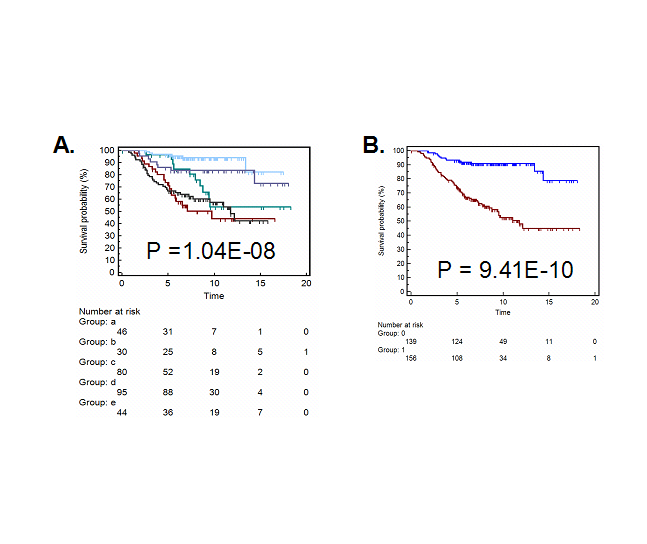

Supplement: Figure S1 — Kaplan-Meier curves of the individual genes that did not accurately predict patient prognosis (p>0.05). A. Results for individual genes in the dataset of Wang, et al. using patient relapse as the endpoint. B. Results for individual genes in the dataset of Sorlie, et al. using patient survival as the endpoint. (0.10 MB TIF) [file pone.0002994.s003.tif]
